# Successful Perioperative Management of Cochlear Implantation in a Patient With Mitochondrial Encephalopathy, Lactic Acidosis, and Stroke-Like Episodes (MELAS)

**DOI:** 10.7759/cureus.27761

**Published:** 2022-08-07

**Authors:** Takumi Kishida, Yusuke Ishida, Toshio Okada, Yumi Tsuzuki, Kenji Kurita, Hiroyuki Uchino

**Affiliations:** 1 Department of Anesthesiology, Tokyo Medical University Hospital, Tokyo, JPN

**Keywords:** mitochondria, cochlear implantation, desflurane, lactic acidosis, mitochondrial encephalopathy

## Abstract

Mitochondrial encephalopathy, lactic acidosis, and stroke-like episodes (MELAS) is a type of mitochondrial disease that is characterized by stroke-like seizures. For these patients, serious, unexpected complications have occurred during and following anesthetic exposure. Provision of anesthesia is challenging, including the choice of anesthetic agents. We here report a case of general anesthesia management for a patient with MELAS. A 46-year-old woman was diagnosed with MELAS at the age of 40. She subsequently underwent cochlear implantation for hearing loss. Anesthesia was induced with midazolam and maintained with desflurane. In the present case, anesthesia was maintained with inhalation anesthetics to avoid the development of propofol infusion syndrome. Her intraoperative and postoperative courses were uneventful. The anesthesia management of patients with MELAS can be performed safely with carefully planned anesthesia and close monitoring at each step, including the postoperative period.

## Introduction

Mitochondrial diseases are congenital mitochondrial genetic disorders, with an estimated incidence of one per 4,000 persons [1]. Mitochondria are the main organelles involved in the electron transport system and oxidative phosphorylation. Patients with mitochondrial diseases develop neurological symptoms, muscle weakness, and/or cardiomyopathy owing to insufficient energy production caused by dysfunction of the electron transport system [1]. There are three major types of mitochondrial diseases, namely, mitochondrial encephalopathy, lactic acidosis, and stroke-like episodes (MELAS), chronic progressive external ophthalmoplegia, and myoclonic epilepsy with ragged-red fibers. Among them, MELAS is characterized by stroke-like episodes [2]. For these patients, serious, unexpected complications have occurred during and following anesthetic exposure. This makes anesthetic management challenging, including the choice of anesthetic agents. Here, we report a case of a patient with MELAS in whom general anesthesia was provided for cochlear implantation.

## Case presentation

A 46-year-old woman (height: 154 cm; weight: 30.3 kg; body mass index: 12.8 kg/m^2^) was diagnosed with MELAS when she was 40 years old. The patient had MELAS due to a point mutation in mitochondrial DNA. She developed hearing loss since her late 30s, and she has been using a hearing aid. Her hearing loss further progressed, and we diagnosed the hearing loss to be due to the progression of MELAS, and cochlear implantation was scheduled. She had a history of diabetes mellitus and epilepsy and was taking oral medications such as levetiracetam, clonazepam, eperisone, and L-arginine. Also, she had been administered subcutaneous insulin (three units in the morning, three units in the afternoon, and five units in the evening). Her preoperative blood examination showed normal hepatic and renal function and a normal creatine phosphokinase (CPK) level, whereas her serum lactate level was high (4.8 mmol/L). While premature ventricular contractions were observed on electrocardiography, on echocardiography there was a normal left ventricular ejection fraction (LVEF) at approximately 60% without abnormal wall motion. Respiratory function tests demonstrated restricted ventilatory impairment with a vital capacity (VC) of 1.5 L and %VC of 39% and forced expiratory volume in one second of 1.15 L and forced vital capacity ratio of 95%. Her general anesthesia plan included avoidance of extended fasting conditions; maintenance of an adequate oxygen supply-demand balance; taking precautions to avoid malignant hyperthermia; and metabolic acidosis that may occur owing to an increased serum lactate level.

**Figure 1:**
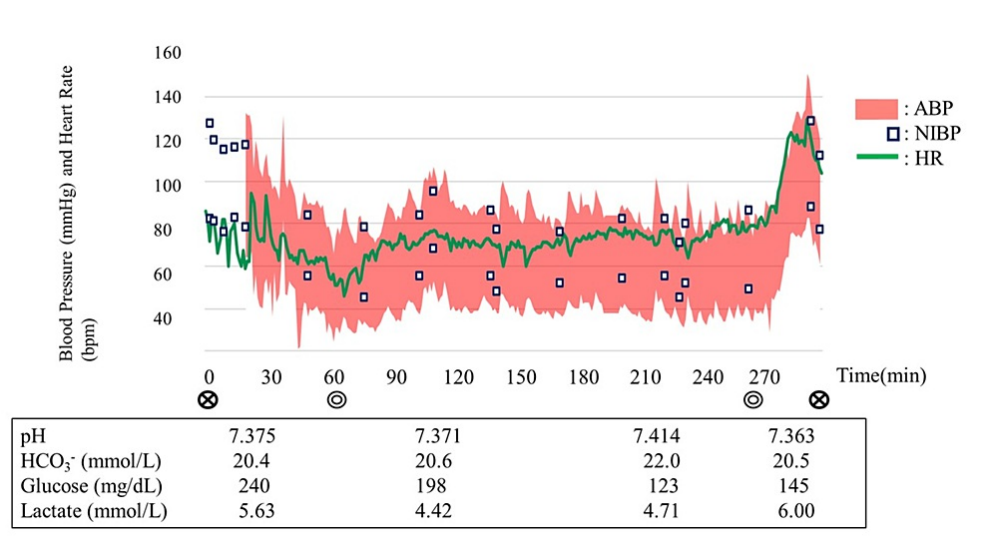
Course of anesthesia of the patient during surgery. ABP: arterial blood pressure, NIBP: non-invasive blood pressure, HR: heart rate.

For anesthesia induction, 3 mg midazolam, 3% desflurane, 100 μg fentanyl, and 15 mg rocuronium were used. During the maintenance of anesthesia, the oxygen concentration was 50%, and the desflurane concentration was adjusted by monitoring the patient's state index and electroencephalogram (EEG) measured using a Sedline® monitor (Masimo Co., Irvine, CA, USA). Remifentanil was also administered at a dose of 0.1 μg/kg/min, and when necessary, additional rocuronium was administered according to the results of neuromuscular monitoring (TOF-Watch® SX; Organon Ireland, Ireland). We did not observe a reduction in regional oxygen saturation (rSO_2_) levels or abnormal EEG during surgery. At the time of anesthesia induction, blood gas analysis demonstrated mild metabolic acidosis with a pH of 7.38, base excess (BE) at −3 mEq/L, and a serum lactate level of 5.6 mmol/L. Although her serum lactate level was high, it did not increase significantly during the surgery. The intraoperative body temperature ranged between 36.5 °C and 37.4 °C. Figure 1 shows the general anesthesia course of the patient during surgery.

Bicarbonated Ringer's solution containing glucose was used as the infusion solution. No adverse events occurred during the surgery. After the surgery, sugammadex was administered to antagonize the muscular relaxant, and the endotracheal tube was extubated before the patient was transferred to the intensive care unit (ICU). The duration of surgery was three hours and 25 minutes, and the duration of anesthesia was five hours and six minutes. Abnormal increases in body temperature and changes in respiratory conditions were not observed during or after the surgery. The administration of L-arginine was resumed immediately to prevent stroke-like episodes. The patient’s postoperative course was favorable, and she was discharged from the hospital on postoperative day four.

## Discussion

Mitochondrial diseases, including MELAS, affect organs with a high oxygen demand, such as the brain, skeletal muscle, and myocardium, owing to disturbance of aerobic energy metabolism caused by mitochondrial dysfunction [1]. Patients with mitochondrial diseases have a potentially high risk of perioperative complications such as respiratory suppression, cardiac dysfunction, arrhythmias, metabolic abnormalities, and/or serious neurological disturbance, due to anesthetic exposure [3]. Therefore, a detailed anesthetic plan, including the anesthetic method, selection of anesthetic drugs and fluid therapy, their dosages, and intraoperative and postoperative management, is necessary prior to the surgical intervention.

First, the number of patients demonstrating worsening of perioperative lactic acidosis upon the use of inhalation anesthetics was reported to be higher than those using propofol anesthesia [4,5]. On the other hand, studies have also reported that the pathogenesis of propofol infusion syndrome (PRIS) is associated with the mitochondrial respiratory chain [6]. It remains unknown whether general anesthesia using intravenous propofol for patients with mitochondrial diseases increases the risk of PRIS [4]. In fact, a survey of pediatric anesthesiologists in the United States showed that approximately 80% used inhalation anesthetics to induce and maintain anesthesia in children with mitochondrial disease [7]. For the reason, we then selected desflurane, an inhaled anesthetic, instead of intravenous propofol anesthesia. We also used midazolam, which is considered to have no suppressive effects on the mitochondrial respiratory chain [8]. Our patient showed no significant progression of metabolic acidosis during or after the surgery.

Second, regarding the use of muscle relaxants, previous reports showed a risk of increased and prolonged effects of muscle relaxants in patients with mitochondrial diseases [9-11]. In the present case, we used a neuromuscular monitor to maintain an adequate muscle relaxation state in the patient during the surgery. Monitoring of muscle relaxation enabled us to avoid the excess administration of muscle relaxants during the surgery, and muscle relaxation was subsequently completely antagonized using sugammadex. The patient was then safely extubated. Considering the risk of recurarization, the patient was transferred to the ICU after surgery to monitor her respiratory state.

Third, regarding the choice of infusion in the perioperative period, in patients with mitochondrial diseases, the tricarboxylic acid cycle is impaired due to mitochondrial abnormalities. Therefore, lactate or acetic acid loading by infusion should be avoided, as they induce acidosis [5,12]. In the present case, we did not use a Ringer solution containing lactate or acetic acid. Intraoperative hypoglycemia in MELAS patients increases the risk of convulsions and stroke-like attacks. In the present case, we used a 5% glucose solution prior to surgery and a bicarbonate Ringer solution containing 2% glucose during the surgery. In MELAS patients, electrolyte abnormalities, including hyponatremia and/or hypokalemia, tend to occur during the perioperative period [13]. Therefore, blood gas analysis was carried out whenever it was necessary to monitor pH, glucose level, and electrolytes, which enabled us to perform safe and adequate perioperative management of the patient.

## Conclusions

Here we reported a case of a patient with MELAS, who underwent successful cochlear implantation without any major complications, as a result of a careful preoperative anesthesia plan and adequate intraoperative anesthetic management. It is important to have sufficient knowledge regarding the possible complications and their causes when performing anesthetic management of MELAS patients, and to pay close attention to the prevention of such complications.
